# Construction Notes

**Published:** 1894-06-23

**Authors:** 


### CONSTRUCTION NOTES.

**New Wing of the New Brighton Convalescent Home.**

This addition to the existing building has lately been opened by the Countess Grosvenor. It is intended to provide private sitting-room and bed-room accommodation for first-class patients. The alteration and enlargement of the home has been superintended by Mr. John Clarke, architect, Castle Street, Liverpool. In this new building are matron's office, reception rooms, 20 bed-rooms, sitting-rooms, and in the basement a spacious recreation hall to accommodate 300 persons. The committee have purchased a large area of land adjoining the building, which will be laid out with tennis-court, lawn, &c.

